# Vision-Ray-Calibration-Based Monocular Deflectometry by Poses Estimation from Reflections

**DOI:** 10.3390/s25154778

**Published:** 2025-08-03

**Authors:** Cheng Liu, Jianhua Liu, Yanming Xing, Xiaohui Ao, Wang Zhang, Chunguang Yang

**Affiliations:** 1 School of Mechanical Engineering, Beijing Institute of Technology, Beijing 100081, China; 15033958125@163.com (C.L.); jeffliu@bit.edu.cn (J.L.); z_w_0112@163.com (W.Z.); 2 State Key Laboratory of Special Vehicle Design and Manufacturing Integration Technology, Baotou 014030, China; su_phoenix@163.com (Y.X.); chunguang33@126.com (C.Y.); 3 Hebei Key Laboratory of Intelligent Assembly and Detection Technology, Tangshan Research Institute, Beijing Institute of Technology, Tangshan 063000, China

**Keywords:** specular surface, deflectometry, vision ray calibration, slope distribution, integral reconstruction

## Abstract

A monocular deflectometric system comprises a camera and a screen that collaboratively facilitate the reconstruction of a specular surface under test (SUT). This paper presents a methodology for solving the slope distribution of the SUT utilizing pose estimation derived from reflections, based on vision ray calibration (VRC). Initially recorded by the camera, an assisted flat mirror in different postures reflects the patterns displayed by a screen maintained in a constant posture. The system undergoes a calibration based on the VRC to ascertain the vision ray distribution of the camera and the spatial relationship between the camera and the screen. Subsequently, the camera records the reflected patterns by the SUT, which remains in a constant posture while the screen is adjusted to multiple postures. Utilizing the VRC, the vision ray distribution among several postures of the screen and the SUT is calibrated. Following this, an iterative integrated calibration is performed, employing the calibration results from the preceding separate calibrations as initial parameters. The integrated calibration amalgamates the cost functions from the separate calibrations with the intersection of lines in Plücker space. Ultimately, the results from the integrated calibration yield the slope distribution of the SUT, enabling an integral reconstruction. In both the numeric simulations and actual measurements, the integrated calibration significantly enhances the accuracy of the reconstructions when compared to the reconstructions with the separate calibrations.

## 1. Introduction

Avoiding damage to the specimens, such as a coordinate measurement machine (CMM) scratching the surfaces [1], noncontact topography measurement methods are widely researched and employed. According to the types of the surface under test (SUT), noncontact methods are generally categorized as methods for diffused SUTs and specular SUTs. Mature fringe projection techniques are typical methods for the reconstruction of diffused SUTs, which complete the reconstruction of SUTs with projectors and cameras [2]. For specular SUTs, interferometry can accurately reconstruct SUTs with simple surfaces like planes and spheres, but fails with SUTs of steep slopes and large size [3]. Overcoming the disadvantages of interferometry, phase measuring deflectometry (PMD) has been extensively researched, with its advantages of full-field measurement capabilities, user-friendly hardware, and noncontact operation [4,5,6]. In recent research, PMD reached the same accuracy of reconstruction with interferometry in the reconstructions of flat mirrors and concave off-axis parabolic mirrors [7]. A PMD system generally consists of cameras and liquid crystal display (LCD) screens. Before measuring the SUT, the system undergoes calibration to define the spatial relationships among the cameras and the screens. During the measurement, the screens display patterns of structured light, while the cameras capture the reflected patterns from the SUT. With the reflected patterns in conjunction with the established spatial relationship, the SUT can be reconstructed in a three-dimensional (3-D) coordinate system.

The precision of PMD is significantly influenced by the precision of the system calibration [8,9,10]. Most research on the calibration is grounded in the pinhole model with distortion [11]. In an ideal pinhole model, every chief ray reaching the image sensor chip of the camera traverses the optical center, with the distance from the optical center to the chip representing the focal length. To address the noticeable deviations between the ideal pinhole images and the actual distorted images, numerous distortion models have been employed to accurately account for these deviations [12,13], such as the models of radial distortion, eccentric distortion, etc. Nevertheless, the multiple sources of error arising from the production and assembly of optical lenses prevent a complete compensation for the deviations, leading to variations in the focal length and distortion on a pixel-by-pixel or piecewise basis.

Vision ray calibration (VRC) significantly enhances the calibration precision of optical instruments such as cameras and projectors. VRC is a calibration technique that does not rely on the direction of the chief ray once it passes through the lens, making it independent of the distortion models. Since each pixel of a camera is linked to a specific chief ray, VRC aims to identify the distribution of these rays, which includes their directions and the 3-D points they traverse before reaching the camera lens [14]. VRC is being extensively employed because of its model-free benefits. Ref. [15] utilizes VRC in multi-camera systems, providing a holistic method of camera system calibration. In [15], an LCD screen serves as an active calibration target, displaying structured light patterns in various positions. The calibration process is finalized through numerical optimization, which guarantees the spatial alignment of the screen points captured by a camera pixel. With a telecentric camera metrology system, Refs. [16,17] utilize VRC in the wavefront reconstruction of the transparent optical components. In [18], VRC is utilized to calibrate stereoscopic PMD (stereo-PMD), addressing the vision ray distribution and the spatial geometric relationship among the cameras and the screen. An in-situ VRC-based stereo-PMD system is developed to reconstruct off-axis aspheric mirrors [19]. The topic ‘VRC-based implementation in real-time and high-speed systems’ is still a challenge, which lies in calibrating with fewer postures of the target and obtaining high accuracy.

Based on the quantity of cameras in PMD, there are two types: multiple-view PMD and monocular PMD (mono-PMD). The multiple-view PMD, which includes more than one image sensor, assesses the slope distribution of an SUT and subsequently performs an integral reconstruction of the SUT [20]. Mono-PMD can reconstruct the SUT using fewer image sensors, but it necessitates precise hardware translation or additional screens in comparison to multiple-view PMD [21,22]. Previous pinhole-model-based mono-PMDs reconstruct SUT by formulating the 3-D coordinate [23], iteratively solving control points of the fitted model [18], or analyzing differential properties of SUT [24]. By combining fringe projection [2] and mono-PMD [25], Ref. [26] effectively measures diffused/specular surfaces. With an infrared projector projecting fringe patterns onto a ground glass, an infrared-PMD is developed to measure discontinuous SUTs [27]. Ref. [3] provides a more detailed review of mono-PMD and analyzes the advantages and disadvantages of several kinds of mono-PMD. In mono-PMD, Refs. [28,29] integrate the processes of calibration and reconstruction into a unified framework. By solving two nonlinear equation systems, Ref. [28] solves relative postures among the screen in several unknown postures and the postures between the camera and the screen. Ref. [29] proposes modal phase measuring deflectometry (MPMD), which models the SUT and makes it possible to simultaneously reconstruct the SUT and calibrate the mono-PMD. However, there will be a pose ambiguity between the screen and the camera when a plane or plane-like mirror is measured. In the measurement of the planar SUTs, with the fixed postures between the reflected virtual camera and the screen, there will be different postures between the planar mirror and the camera to generate the virtual camera, resulting in pose ambiguity. The methods in Refs. [28,29] are not appropriate for planar and plane-like SUTs.

To improve the accuracy and reliability of the mono-PMD, this paper proposes a VRC-based mono-PMD. It involves two VRC-based separate calibrations to individually calibrate the PMD. With the initial values solved in the separate calibrations, an integrated calibration is performed, which combines the separate calibrations and the quantitative criterion of the intersection of lines. An integral reconstruction is followed to reconstruct the SUT. The integrated calibration combines the PMD calibration and the SUT measurement into a cost function. Meanwhile, it resolves the pose ambiguity in the measurement of the planar and plane-like SUTs through the integrated calibration.

This paper is organized as follows: In Section 1, deflectometry and vision ray calibration are reviewed; in Section 2, the VRC-based mono-PMD is detailed; Section 3 presents both simulated and real experiments to validate the proposed approach; finally, Section 4 offers conclusions and future directions of research.

## 2. Proposed Methodology

The proposed VRC-based mono-PMD involves the VRC-based calibration of the PMD and the integral reconstruction of the SUT. In Section 2.1, the VRC algorithm is briefly formulated. The proposed calibration consists of two distinct steps for separate calibrations (Section 2.2 and Section 2.3) and a third step for integrated calibration (Section 2.4). With an assisted flat mirror, the first step is to solve the ray distribution of the camera and the relative postures between the screen and the camera. The second step is to solve the ray distribution of the SUT. In the third step, the cost function of the spatial line intersection and the cost functions in the first and second steps are integrated into one cost function. With the previously solved postures as the initial values, the ray distributions of the camera and the SUT, as well as the relative posture, are solved by minimizing the integrated cost function. With the solved parameters, the slope distribution of the SUT is calculated to reconstruct the SUT integrally. This section outlines the algorithm for the VRC, followed by the calibrations for the mono-PMD.

### 2.1. Vision Ray Calibration

**Figure 1:**
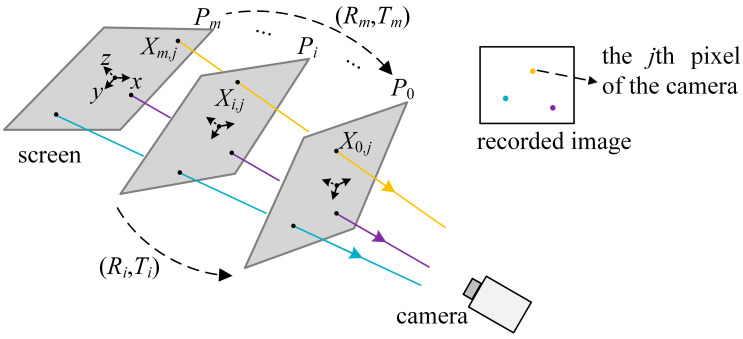
Diagram of the VRC.

Illustrated in Figure 1, in the posture *P_i_* (*i* = 0, …, *m*) with the rotation matrix *R_i_* and the translation vector *T_i_* (the dashed arrows in Figure 1) to *P*_0_, the screen with a height of *h* and a width of *w* displays the phase-shifted [30] horizontal and vertical fringe patterns with the fringe orders *N*^2^, *N*^2^−1, *N*^2^−*N* selected by the optimum fringe frequency method [31]. From the recorded images of the camera, the extracted phase provides the dense correspondence between the pixels of the camera and the screen. For the *j*th (*j* = 1, …, *n*) camera pixel with the recorded horizontal and vertical phase values $\varphi_{h,i}$ and $\varphi_{v,i}$ in *P_i_* of the screen, the corresponding screen pixels *X_i_*_,*j*_ (*x*, *y*, 0) are spatially collinear, where

(1) $$x=-\varphi_{v,i}w/\left(2\pi N^{2}\right),\;y=-\varphi_{h,i}h/\left(2\pi N^{2}\right).$$

As *X_i_*_,*j*_ is transferred to the coordinate system (CS) of *P*_0_, it is centered as

(2) $$Xcenter_{i,j}=R_{i}X_{i,j}+T_{i}-\sum_{i=0}^{m}\left(R_{i}X_{i,j}+T_{i}\right)/\left(m+1\right),$$

the direction of the fitted chief ray solved by the centered *X_i_*_,*j*_ is denoted as (*um_j_*, *vm_j_*, 1)*^T^* where

(3) $$um_{j}={\sum_{i=0}^{m}\left(Xcenter_{i,j}\left(1\right)Xcenter_{i,j}\left(3\right)\right)/\sum_{i=0}^{m}Xcenter_{i,j}\left(3\right)}^{2},\;vm_{j}={\sum_{i=0}^{m}\left(Xcenter_{i,j}\left(2\right)Xcenter_{i,j}\left(3\right)\right)/\sum_{i=0}^{m}Xcenter_{i,j}\left(3\right)}^{2}.$$

Thus, the coordinate deviations between *Xcenter_i_*_,*j*_ and the point of the same *z* component on the fitted chief ray are expressed as

(4) $$\Delta x_{i,j}=Xcenter_{i,j}\left(1\right)-Xcenter_{i,j}\left(3\right)\cdot um_{j},\;\Delta y_{i,j}=Xcenter_{i,j}\left(2\right)-Xcenter_{i,j}\left(3\right)\cdot vm_{j}.$$

To solve the relative postures among *P_i_* and *P*_0_, the cost function,

(5) $$f\left[R_{i}\left(\alpha_{i},\;\beta_{i},\;\gamma_{i}\right),\;T_{i}\right]=\sum_{j=1}^{n}\sum_{i=0}^{m}\left(\Delta x_{i,j}^{2}+\Delta y_{i,j}^{2}\right)$$

is minimized to solve the relative postures using the Levenberg–Marquardt (LM) algorithm [32], with the initial values solved by the ideal pinhole model [11], where $\alpha_{i}$, $\beta_{i}$ and $\gamma_{i}$ denote the rotation angles around *x*, *y* and *z* axes from the CS of *P_i_* to *P*_0_, respectively. With the solved *R_i_* and *T_i_*, any points on the chief ray of the *j*th camera pixel are expressed as

(6) $$l_{j}=X_{0,j}+k\cdot {\left(um_{j},\;vm_{j},\;1\right)}^{T}$$

where *k* is a scale factor. For the movement of the screen along the *z* direction of the screen CS, Equations (3), (4) and (6) are scaled by the *z* component of the centered coordinates.

### 2.2. Vision Ray Calibration of the Camera in Monocular Deflectometry

**Figure 2:**
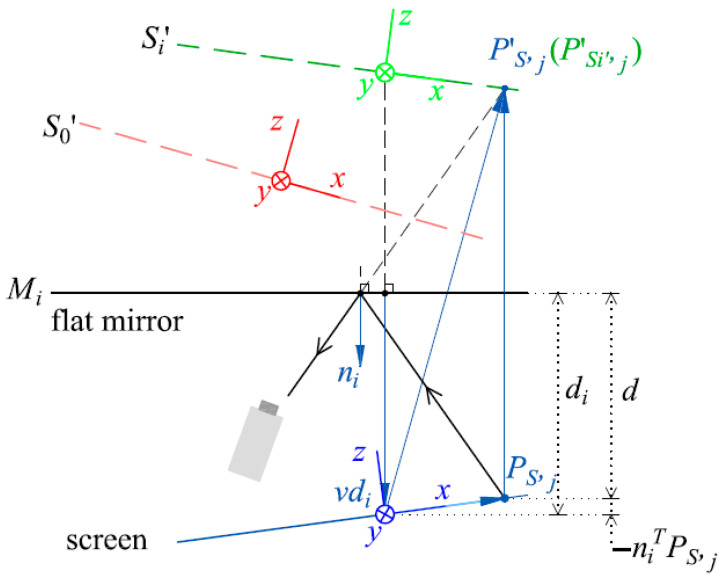
Schematic diagram of the VRC-based PMD calibration.

The first step of the mono-PMD calibration is to solve the vision ray distribution of the camera and the relative postures among the screen and the reflected virtual screens. Illustrated in Figure 2, the camera records the virtual screens *S_i_*′ reflected by an assisted flat mirror in the various postures *M_i_* (*i* = 0, …, *m*) while the stationary screen displays the fringe patterns.

In the CS of the screen, the distance between the flat mirror and the origin of the screen CS along the normal direction *n_i_* of the flat mirror is denoted as *d_i_*. In the CS of the screen, recorded by the *j*th camera pixel, a point *P_S_*_,*j*_ is located at a distance *d* to *M_i_* along *n_i_*. As a virtual image of *P_S_*_,*j*_, the point is denoted as *P*′*_S_*_,*j*_ and *P*′*_Si_*_′,*j*_ in the CSs of the screen and *S_i_*′, respectively. According to the geometry of the mirror reflection, there are

(7) $$\left\{\begin{array}{l} d=d_{i}+n_{i}^{T}P_{S,j}\\ {P^{\prime }}_{Si^{\prime },j}=(I-2ee^{T})P_{S,j}\\ {P^{\prime }}_{S,j}=P_{S,j}-2dn_{i}=R_{Si^{\prime }\_S}{P^{\prime }}_{Si^{\prime },j}+T_{Si^{\prime }\_S} \end{array}\right.,$$

where *R_Si_*_′_*S*_ and *T_Si_*_′_*S*_ denote the rotation matrix and the translation vector from the CS of *S_i_*′ to the CS of the screen, respectively; *e* denotes [0 0 1]*^T^*. Derived from Equation (7), the rotation matrix and the translation vector from the CS of the screen to the CS of *S_i_*′, *R_S_*__*Si*′_ and *T_S_*__*Si*′_, are formulated as

(8) $$\left\{\begin{array}{l} R_{S\_Si^{\prime }}=(I-2ee^{T})(I-2n_{i}n_{i}^{T})\\ T_{S\_Si^{\prime }}=2R_{S\_Si^{\prime }}d_{i}n_{i} \end{array}\right..$$

From Equation (8), *R_Si_*_′_*S*0′_ and *T_Si_*_′_*S*0′_ from the CS of *S_i_*′ to the CS of *S*_0′_ are formulated as

(9) $$\left\{\begin{array}{l} R_{Si^{\prime }\_S0^{\prime }}=R_{S\_S0^{\prime }}(I-2n_{i}n_{i}^{T})(I-2ee^{T})\\ T_{Si^{\prime }\_S0^{\prime }}=-2R_{S\_S0^{\prime }}d_{i}n_{i}+T_{S\_S0^{\prime }} \end{array}\right..$$

*R_Si_*_′_*S*0′_ and *T_Si_*_′_*S*0′_ can be estimated initially with the calibration algorithm of the pinhole model. The eigenvector of (*I*−2*ee^T^*)*R_Sj_*_′__*_S_*_0′_*R_Si_*_′_*S*0′_(*I*−2*ee^T^*) (*i* ≠ *j*) corresponding to the eigenvalue 1 is in the direction of *n_i_* × *n_j_*. *n_i_* is estimated as (*n_i_* × *n_j_*) × (*n_i_* × *n_k_*) (*I* ≠ *j* ≠ *k*). Substitute *n_i_* into Equation (8), *R_S_*__*Si*′_ and *T_S_*__*Si*′_ are solved. With the estimated *n_i_*, *R_S_Si_*′ and *T_S_Si_*′, *T_Si_*_′_*S*0′_ in Equation (9) provides a system of linear equations to solve *d_i_*. With the solved initial values of *n_i_* and *d_i_*, *R_i_* and *T_i_* in the cost function of Equation (5) are substituted by *R_Si_*_′_*S*0′_ and *T_Si_*_′_*S*0′_ in Equation (9), respectively. In the CS of *S_i_*′, *X_i_*_,*j*_ is obtained by substituting the extracted phase values from the recorded fringe patterns into Equation (1). *vd_i_* denotes that *d_i_*·*n_i_* decreases the number of parameters in the cost function

(10) $$f_{camera}\left[vd_{i}\right]=\sum_{j=1}^{n}\sum_{i=0}^{m}\left(\Delta x_{i,j}^{2}+\Delta y_{i,j}^{2}\right),$$

which is obtained by substituting Equation (9) into Equation (5).

By minimizing the cost functions in Equation (10) with the LM algorithm, *vd_i_* is solved to calculate *R_S_S_*_0′_ and *T_S_S_*_0′_ and to calculate the vision ray distribution of the camera with Equation (6).

### 2.3. Vision Ray Calibration of the SUT in Monocular Deflectometry

**Figure 3:**
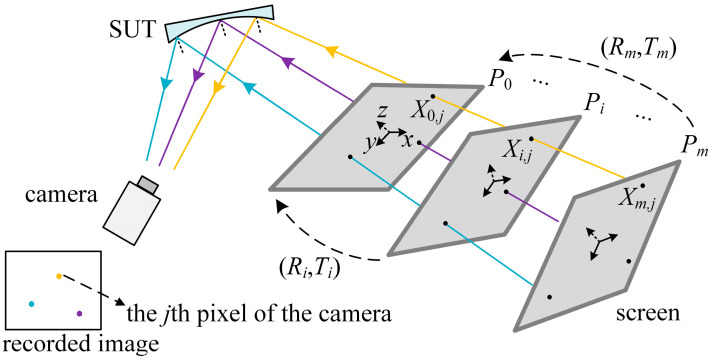
Diagram of the VRC of the SUT.

The second step of the calibration is to solve the vision ray distribution of the SUT. The vision rays are between the SUT and the screen. Illustrated in Figure 3, in which a SUT is measured after the first calibration step, the camera records the reflected fringe patterns by the SUT while the screen displays the fringe patterns in several different postures *P_i_* (*I* = 0, …, *m*), and *P*_0_ maintains the posture in the first step in Section 2.2.

In this step, with the recorded reflected fringe patterns, the coordinate of *X_i_*_,*j*_ (*x*, *y*, 0) in the CS of the *P_i_* is calculated by Equation (1). Through Equations (2) and (4), the relative postures *R_i_* and *T_i_* of the different postures of the screen are iteratively solved by minimizing Equation (5), which is denoted as *f_SUT_* in this step, with the LM algorithm. The distribution of the vision ray is solved by Equation (6). In confirming the initial values of the iteration for the planar and plane-like SUTs, it is obtained in the pinhole-model camera calibration with the virtual screen as a phase target; for the curved SUTs, the initial values can be obtained by the movement estimation of the equipment jointing the screen, such as a robot arm, translating stage, etc.

### 2.4. Integrated Vision Ray Calibration of Monocular Deflectometry

**Figure 4:**
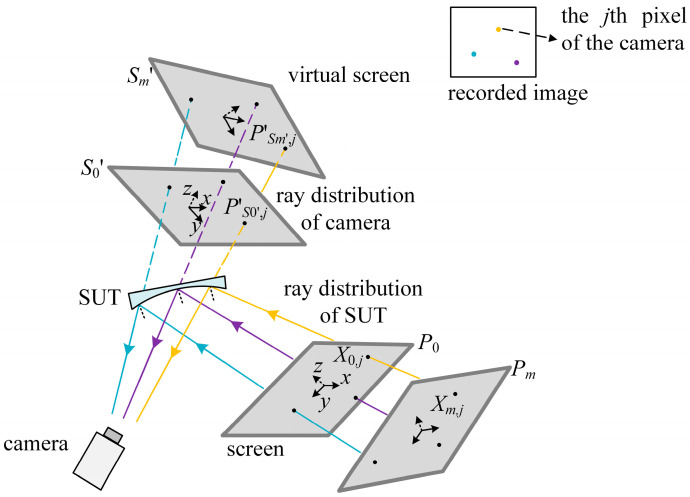
Diagram of the integrated VRC.

As illustrated in Figure 4, for every camera pixel, the vision rays solved in the first and second steps should intersect with each other at the point of reflection. Disturbed by electronic noise, the rays do not meet. To improve the accuracy of the calibration by reducing the distance of the rays corresponding to the same camera pixel, the cost functions in the first and second steps are integrated along with the concept of the spatial line intersection in Plücker space.

A spatial line passing through the points *P*(*p_x_*, *p_y_*, *p_z_*) and *Q*(*q_x_*, *q_y_*, *q_z_*) is expressed as a Plücker vector

(11) $$L=\left(\begin{array}{l} l_{0}\\ l_{1}\\ l_{2}\\ l_{3}\\ l_{4}\\ l_{5} \end{array}\right)=\left(\begin{array}{c} p_{x}q_{y}-q_{x}p_{y}\\ p_{x}q_{z}-q_{x}p_{z}\\ p_{x}-q_{x}\\ p_{y}q_{z}-q_{y}p_{z}\\ q_{y}-p_{y}\\ p_{z}-q_{z} \end{array}\right).$$

For two lines of the Plücker vectors L and V, the lines intersect with each other if and only if

(12) $$f_{line}=l_{0}v_{5}+l_{1}v_{4}+l_{2}v_{3}+l_{3}v_{2}+l_{4}v_{1}+l_{5}v_{0}=0.$$

In the integrated calibration of the mono-PMD, recorded by the *j*th camera pixel, the points on *S*_0′_ and *S_m_*′ in the first step, the points on *P*_0_, *P_m_* in the second step are selected to calculate the Plücker vectors $L_{j}$ and $V_{j}$. Before the calculation of $L_{j}$ and $V_{j}$, the points on *S_m_*′, *P*_0_ and *P_m_* are transformed to the CS of *S*_0′_ with *R_Sm_*_′_*S*0′_, *T_Sm_*_′_*S*0′_, *R_S_ S_*_0′_ and *T_S_ S_*_0′_ in Section 2.2 and *R_m_* and *T_m_* in Section 2.3. Integrating the cost functions *f_camera_* and *f_SUT_* in the first and second steps, and the cost function about the intersection of the lines, the integrated cost function

(13) $$g\left[vd_{i},R_{i}\left(\alpha_{i},\;\beta_{i},\;\gamma_{i}\right),\;T_{i}\right]=f_{camera}+f_{SUT}+\lambda \sum_{j=1}^{n}f_{line,j}$$

is minimized with the LM algorithm with the results of the previous calibration as the initial values. In Equation (13), *λ* is a constant weight coefficient to scale *f_line_* to the same numerical magnitude as *f_camera_* and *f_SUT_*. In constructing *f_SUT_*, the direction of the movement of the screen in the CS of *S*_0′_ determines the component used to scale the centered coordinate in Equations (3), (4) and (6).

With the relative postures among *S_i_*′ calculated by Equations (9) and (13) and the relative postures among *P_i_* by Equation (13), the vision ray distributions of the camera and the SUT are solved by Equation (6) in the CSs of *S*_0′_ and *P*_0_, respectively. With the relative postures between *S*_0′_ and *P*_0_ calculated by Equations (8) and (13), the ray distribution of the SUT is transformed to the CS of *S*_0′_ to calculate the middle point of the common vertical line of the lines $L_{j}$, $V_{j}$ and their bisectors, which are the approximation of the normal direction of the SUT. With the *x*, *y* components of the middle points and the slope distribution of the SUT, the SUT is reconstructed integrally.

## 3. Experiments

### 3.1. Simulation

**Figure 5:**
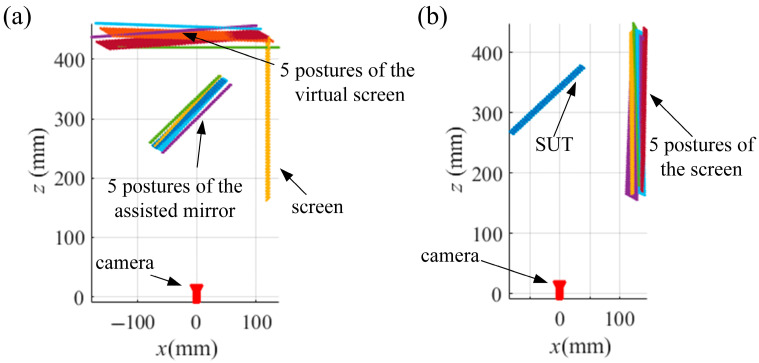
Relative postures among camera, screen and SUT. Relative postures in the calibrations: (**a**) Section 2.2, (**b**) Section 2.3.

To test the proposed VRC-based mono-PMD, some simulated experiments were carried out. As SUTs, a specular cylinder and a specular ball with a radius of 200 mm are measured by a simulated system of the mono-PMD. In the calibration of the system, the assisted flat mirror and the screen are moved into five different postures. The ideal fringe patterns captured by a simulated camera based on the ideal pinhole model are generated with the relative positions among the screen, the camera, and the assisted flat mirror or the SUTs, as depicted in Figure 5. Figure 5a illustrates the five postures of the assisted mirror in the calibration of the camera; Figure 5b illustrates the five postures of the screen in the calibration of the SUT.

Gaussian noises with a standard deviation (std) *σ* from 0 to 2 in increments of 0.2 are added to the ideal fringe patterns. The separate calibrations are conducted with the pixels of an even interval of 20 px in the 1800 × 1600 px central region of the phase maps. The integrated calibration is conducted with the common integer pixels in the separate calibrations. With the noisy patterns generated from the relative postures in Figure 5a, the separate calibration method in Section 2.2 is conducted to calibrate the relative posture between the CSs of the screen and *S*_0′_. With the noisy patterns generated from the relative postures in Figure 5b, the separate calibration method in Section 2.3 is conducted to calibrate the relative postures among the CSs of the screen. With the calibrated relative postures from the separate calculations as initial values, the integrated calibration method in Section 2.4 is conducted to calculate the ray distributions of the camera and the SUT and to solve the slope distributions of the SUTs. The reconstructions of the SUTs are conducted with the integral reconstruction algorithm [33]. Meanwhile, the SUTs are reconstructed with the slope distributions solved from the separate calibrations to compare accuracy with the integrated calibration.

**Figure 6:**
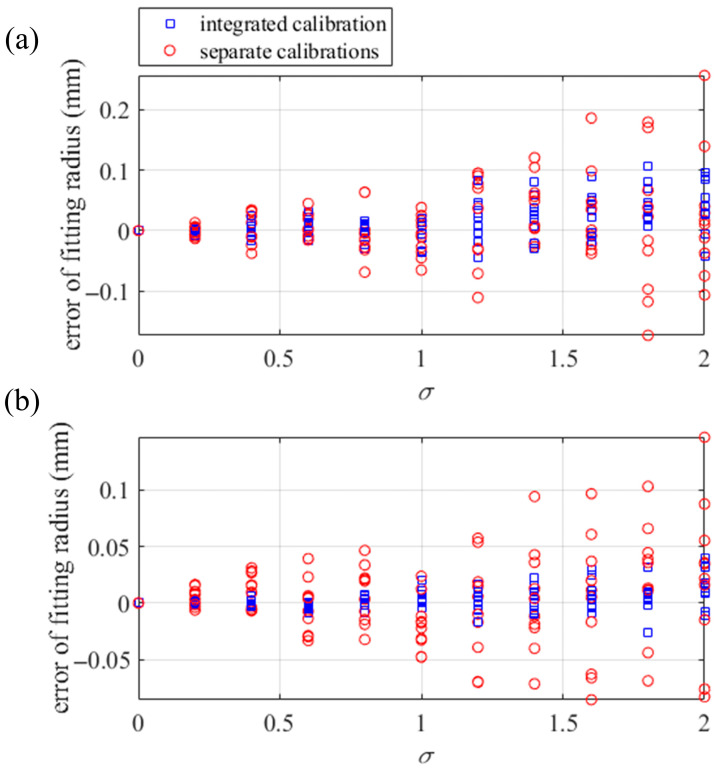
Deviations of the fitting radii. Radius deviations in (**a**) fitting the cylinder and (**b**) fitting the ball.

Under the same std of noise, Gaussian noise is randomly generated ten times to add to the ideal fringe patterns. Under every std of the noise, the calibrations and reconstructions are repeated ten times with the different noisy fringe patterns of the same std. After the reconstructions, the fitting of the cylinder and ball is performed on the reconstructed point clouds of the SUTs. Compared to the real radius, the deviations of the fitting radii are illustrated in Figure 6. The radius deviations of the reconstructed cylinder tend to be higher than those of the ball. The reason for this phenomenon is the different number of parameters used to fit the ball and the cylinder. Fitting the ball involves the radius and the coordinates of the center of the ball, which is a total of four parameters to be solved. In fitting the cylinder, there is the radius of the cylinder, the direction of the axis of the cylinder, and a two-dimensional coordinate where the axis crosses the *xoz* plane of the CS of S_0′_, a total of six parameters to be solved. With the same reconstruction errors, it is acceptable that the fitted cylinder radii are further from the ideal radius than the fitted ball radii.

The results of the simulated experiments indicate that the integrated calibration is of high noise resistance and high reconstruction accuracy. Compared to the separate calibrations, the integrated calibration can effectively improve the calibration accuracy of the mono-PMD.

### 3.2. Actual Measurement

**Figure 7:**
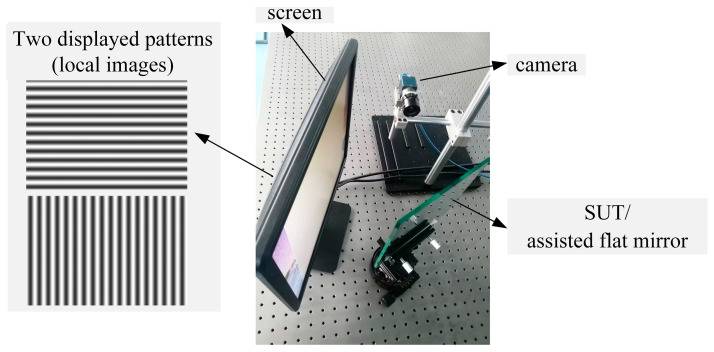
Hardware diagram of the mono-PMD system.

The deflectometric system consisted of a screen and a camera, as depicted in Figure 7. The camera (manufacturer: DAHENG IMAGING, Beijing, China; model: MER2-503-36U3 M; resolution: 2048 × 2448; pixel (px) interval: 3.45 μm) matched a standard prime lens (manufacturer: AZURE Photonics, Fuzhou, Fujian province, China; model: AZURE-1614MM; focal length: 16 mm). In the vertical and horizontal directions of the screen, it (manufacturer: Dell Technologies, Round Rock, Texas, USA; model: E1715S; resolution: 1280 × 1024; pixel interval: 0.264 mm) displayed the phase-shifted cosinoidal fringe patterns with optimal fringe orders of 144, 143 and 132.

**Figure 8:**
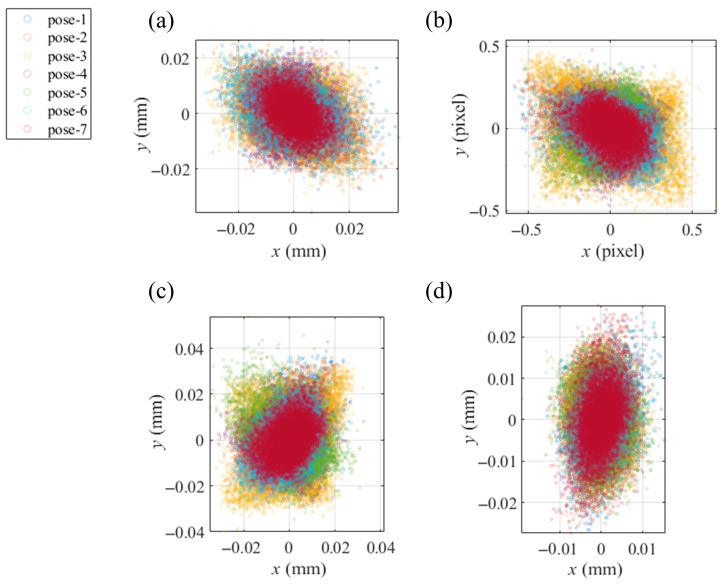
Distributions of the reprojection errors in separate calibrations, where different colors indicate the errors associated with the different postures of the assisted mirror. Reprojection errors of (**a**) the VRC-based calibration of the camera in Section 2.2, (**b**) the PHC-based calibration of the camera on the image sensor, (**c**) the PHC-based calibration of the camera reprojected to the screen, (**d**) the VRC-based calibration of the SUT in Section 2.3.

With the shape deviation from an ideal plane of under 1 μm, a flat mirror in 7 different postures covered the view of the camera and reflected the displayed fringe patterns, which were recorded by the camera. Solved by the techniques of phase extraction, the absolute unwrapping of vertical and horizontal phase maps was extracted from the recorded patterns. From the phase maps, Equation (1) solved the coordinates of the recorded points in the CSs of the virtual screens. In the central region, with 1600 × 1400 px phase maps, 8876 integer pixels, with an even interval of 15 px, were selected to calibrate the camera with the method in Section 2.2, obtaining *vd_i_*, *R_S_*_0′_ and *T_S_*_0′_ as the initial values for the integrated calibration. The iteration processes were performed with a computer (manufacturer: Dell Technologies, Round Rock, Texas, USA; model: OptiPlex 7000; CPU: i7-12700; RAM: 16 GB) and the software MATLAB R2022b Update 10 (9.13.0.2698988), spending 8 iterations and 3.4487 s. With the mean value of [10^−16^, 10^−16^] mm and the std of [0.0074, 0.0065] mm, the distributions of the residual calibration error $\Delta x_{i,j}$ and $\Delta y_{i,j}$ are illustrated in Figure 8a. With the same operation of the hardware, the traditional PMD calibration based on a pinhole camera calibration (PHC) obtained the intrinsic parameters of the camera and the relative postures among the screen, the camera and the assisted mirror.

To test the accuracy of the calibration based on the VRC using the same integer pixels in the phase maps, the PMD calibration based on PHC was conducted [34]. With the mean value of [10^−11^, 10^−12^] px and the std of [0.1422, 0.1233] px, the distribution of the reprojection errors of the PHC on the pixel CS of the camera is illustrated in Figure 8b. To compare the calibration results based on the VRC and the PHC, with the PHC-solved relative postures and the intrinsic parameters, the undistorted integer pixels were reprojected to the plane of the screen with the reprojection errors of the mean value of [10^−7^, 10^−7^] mm and the std of [0.0073, 0.0073] mm illustrated in Figure 8c. Comparing Figure 8a,c, it is evident that the error distribution of the VRC is more tightly clustered around the origin, indicating that the VRC provides a more reliable calibration than the PHC.

After the separate calibration of the camera, a planar mirror with a size of 45 mm × 40 mm as an SUT was measured. The screen displayed the fringe patterns in 7 different postures, while the first posture remained the same as in the calibration of the camera. The SUT was fixed to reflect the patterns that were recorded by the camera. Using the method in Section 2.3, the identical integer pixels from the calibration of the camera were employed to calibrate the SUT, obtaining the relative postures *R_i_* and *T_i_* between the postures of the screen, which served as the initial values for the integrated calibration. The processes took 10 iterations and 5.6343 s. The error distribution in calibrating the SUT is illustrated in Figure 8d.

**Figure 9:**
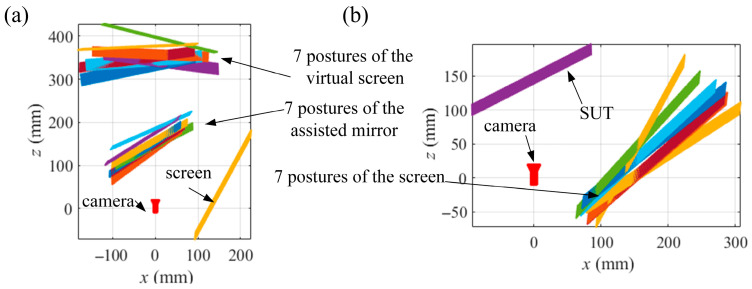
The relative postures of the hardware. The relative postures in (**a**) the calibration of the camera in Section 2.2 and (**b**) the calibration of the SUT in Section 2.3.

**Figure 10:**
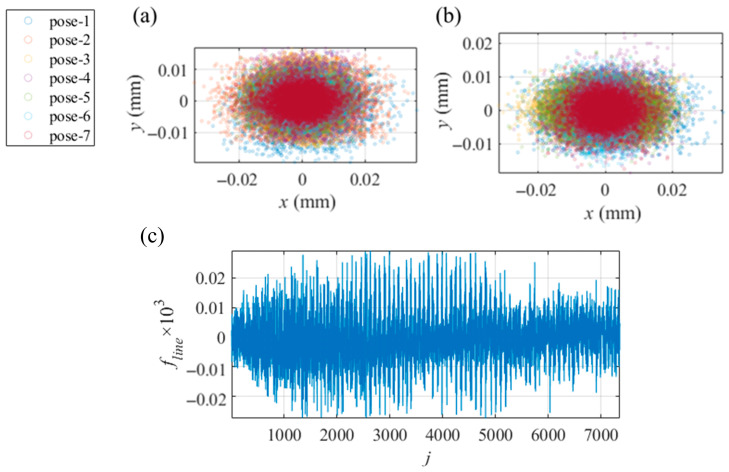
Distribution of the reprojection errors in the integrated calibration, where different colors indicate the errors associated with the different postures of the assisted mirror and the screen. Distribution of the errors of (**a**) the camera, (**b**) the SUT, (**c**) the residual *f_line,j_*.

With the initial values and the pixels corresponding to the error $\sqrt{\Delta x_{i,j}^{2}+\Delta y_{i,j}^{2}}$ exceeding 4 times its mean value filtered, the process of the integrated calibration in Section 2.4 was conducted twice to improve the accuracy of the calibrations. *f_line_*_,*j*_ was computed with two normalized Plücker vectors. The weight coefficient *λ* was set as 10^3^, thereby scaling *f_line_* to the same numerical magnitude as *f_camera_* and *f_SUT_*. Before the second integrated calibration, the pixels corresponding to *f_line_*_,*j*_ exceeding 4 times its mean value were excluded from the calibration, resulting in a total of 7351 remaining pixels. The integrated calibration iteration took 14 iterations and 32.5460 s. After the processes of the integrated calibration, the ray distributions of the camera and the SUT and the relative posture among the screen and its virtual images were the calibration results. Transformed to the CS of the camera for visualization using the posture parameters determined with [34], the relative postures are illustrated in Figure 9. The residual error distributions in the integrated calibration are illustrated in Figure 10.

**Figure 11:**
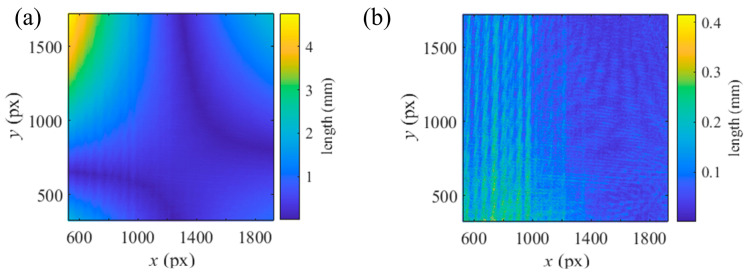
Length of the common vertical line corresponding to integer camera pixels. Length solved with the results of (**a**) the separate calibrations and (**b**) the VRC-based integrated calibration.

**Figure 12:**
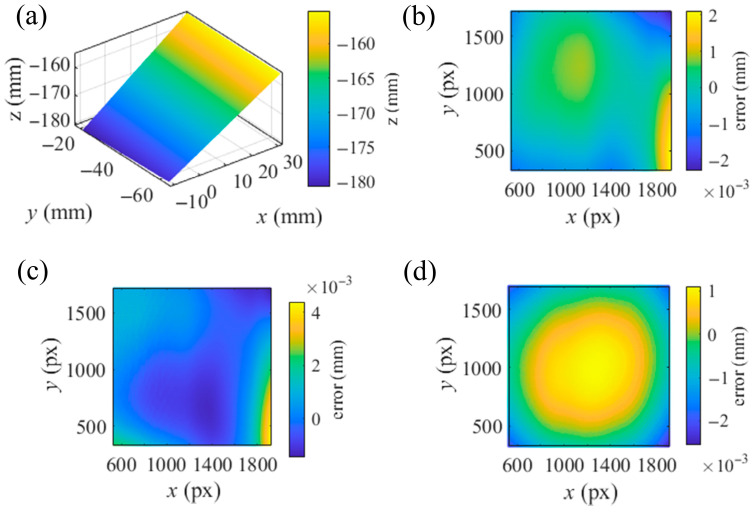
Reconstruction and the residual errors associated with plane fittings. (**a**) Reconstruction with the results of the VRC-based integrated calibration. Residual errors of the reconstruction with the results of (**b**) the VRC-based integrated calibration, (**c**) the VRC-based separate calibrations, (**d**) the PHC-based PMD calibration algorithm and the modified MPMD.

The length of the common vertical line between the camera ray and the SUT ray, with the results of the separate calibrations and the integrated calibration, corresponding to each integer camera pixel, is depicted in Figure 11. With the integrated calibration results, the length of the common vertical lines was reduced from the mean value of 0.8086 mm and the std of 0.8443 mm with the separate calibration results to the mean value of 0.0498 mm and the std of 0.0468 mm. With the slope distribution solved by the ray distributions and the *x*, *y* components of the middle points, the SUT was integrally reconstructed in the CS of *S*_0′_. Figure 12 presents the residual errors in the *z* direction of the CS of *S*_0′_ for each integer pixel of the camera, associated with the plane fitting of the reconstruction. Reconstructed with the results of the separate calibrations and the integrated calibration, the 3-D reconstructions were fitted by planes with root mean errors (rmses) of 8.6356 × 10^−4^ mm and 5.7982 × 10^−4^ mm, respectively. The integrated calibration effectively improves the accuracy of the calibration and the reconstruction. To compare the reconstructions with the calibrations based on the VRC and the PHC, the PHC-based PMD calibration algorithm [34] was conducted with two parameters of tangential distortion and three parameters of radial distortion. A modified MPMD [29] approach was implemented using a cubic B-spline surface with a knot interval of 70 px to model the SUT, along with the results from the PHC-based calibration. This modified MPMD did not involve optimizing the parameters related to the camera and screen positioning. The reconstruction achieved by the modified MPMD was fitted with a plane with an rmse of 7.0182 × 10^−4^ mm towards the fitted plane, as shown in Figure 12d. From the reconstructions of the SUT, the VRC-based calibration and reconstruction are more reliable than those of the PHC.

**Figure 13:**
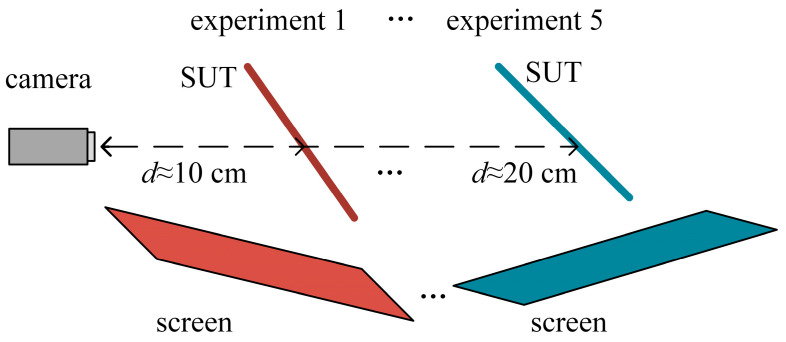
Experiments to test the reliability of the mono-PMD.

To evaluate the reliability of the VRC-based integrated calibration, five experiments were carried out with the distance *d* between the planar SUT and the camera varying from 10 cm to 20 cm in increments of 2.5 cm (Figure 13). Reconstructed with the results of the VRC-based integrated calibration (IC) and separate calibrations (SC), the rmse and the peak–valley value (pv) of the residual errors associated with the plane fitting of the reconstructions are summarized in Table 1. The data presented in the table demonstrates that the VRC-based integrated calibration yields more reliable reconstructions compared to the separate calibrations, while also exhibiting a high degree of repeatability.

**Figure 14:**
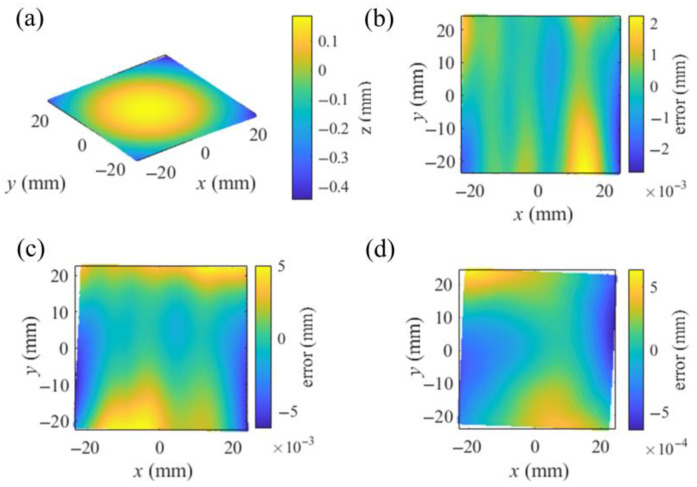
Reconstruction and the residual errors associated with ball fittings. (**a**) Reconstruction with the results of the VRC-based integrated calibration. Residual errors of the reconstruction with the results of (**b**) the VRC-based integrated calibration, (**c**) the VRC-based separate calibrations, (**d**) the PHC-based PMD calibration algorithm and MPMD.

With *d* around 20 cm and the same procedures of calibrations and reconstruction, a 50 × 50 mm spherical concave mirror with a radius of 1000 mm as an SUT was measured. Illustrated in Figure 14, the SUT was reconstructed with separate calibrations (SCs), integrated calibrations (ICs) and MPMD, respectively. The reconstructions were fitted with balls. The fitted radii of the reconstructions, rmse and pv associated with the ball fittings are summarized in Table 2. The reconstruction with IC reached higher accuracy in the fitted radius than with SC and MPMD, while MPMD reached the lowest pv and rmse.

**Figure 15:**
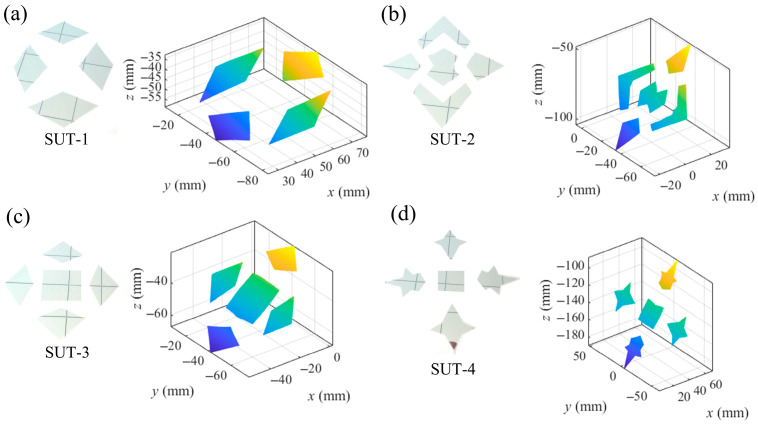
Separate SUTs and the reconstructions. (**a**) SUT-1 and its reconstruction, (**b**) SUT-2 and its reconstruction, (**c**) SUT-3 and its reconstruction, (**d**) SUT-4 and its reconstruction.

To further test the performance of the proposed VRC-based mono-PMD, four SUTs with the coplanar separate flat mirrors illustrated in Figure 15 were measured. The slope distributions of the SUTs were solved by the VRC-based integrated calibration. Describing the height increment of a continuous surface, the reconstruction of every separate mirror with a mean height of zero was conducted by the integral reconstruction algorithm [33]. Every reconstruction of an individual mirror was adjusted based on the mean distance between the middle points and the relative reconstruction corresponding to the same integer pixels. The reconstructions of the SUTs were, respectively, fitted with planes with rmse 0.0029 mm, 0.0066 mm, 0.0053 mm, and 0.0112 mm towards the fitted planes. The proposed VRC-based mono-PMD reaches higher precisions than the PHC-based reconstruction method [35] in the reconstruction of the separate mirrors.

## 4. Conclusions

In this paper, vision-ray-calibration-based monocular phase measuring deflectometry is proposed. The deflectometry integrates the calibration of the system and the measurement of the specular surface with an integrated cost function, which improves the accuracy of the measurement and the calibrations compared to the separate calibrations. With the integration, the proposed method is more robust to the disturbance of hardware, such as camera installation errors or small rotations in the screen’s postures, than the PMD methods, which separate the calibration and the measurement. In the integrated calibration, a flat mirror in several postures assists the calibration; the screen in several postures displays fringe patterns reflected by the SUT. Because the parameters of the movement of the flat mirror and the screen are calculated by the integrated calibration, there is low demand for accuracy in the movements. The pose ambiguity in the measurement of planar and plane-like mirrors is addressed with the integrated calibration. Generally solved with multi-view PMD, the slope distributions of SUT are measured successfully with the proposed method of mono-PMD. Compared to pinhole-model-based deflectometry, vision-ray-calibration-based deflectometry can reach higher accuracy in the reconstructions of the continuous specular surfaces and the separate specular surfaces. In the future, vision-ray-calibration-based deflectometry will match accurate mechanics, such as a robot arm, to improve measurement efficiency and the precision of accurate mechanics; meanwhile, more curved specular surfaces will be measured.

## Figures and Tables

**Table 1:** PV and rmse of the residual errors associated with plane fitting (μm).

*d*	10 cm		12.5 cm		15 cm		17.5 cm		20 cm	
	SC	IC	SC	IC	SC	IC	SC	IC	SC	IC
**rmse**	0.9914	0.3748	1.4031	0.3647	1.2524	0.4657	1.2524	0.4657	2.7807	0.4894
**pv**	6.3432	2.8618	8.2452	2.0960	7.1371	3.0719	7.1371	3.0719	15.5349	4.3552

**Table 2:** Details of the ball fittings (mm).

	SC	IC	MPMD
**fitt** **ed radius**	945.3867	997.9301	994.0202
**rmse**	0.0020	7.0839 × 10^−4^	2.3756 × 10^−4^
**pv**	0.0112	0.0050	0.0013

## Data Availability

Data underlying the results presented in this paper are not publicly available at this time but may be obtained from the authors upon reasonable request.
